# The Transdiagnostic Association Between Childhood Trauma and Accelerated Brain Aging in Two Independent Datasets

**DOI:** 10.1093/schbul/sbaf204

**Published:** 2025-11-23

**Authors:** Lan Zhou, Hugo Schnack, Edwin van Dellen, Marco P Boks, P Roberto Bakker, Wiepke Cahn, Iris E C Sommer, Marieke J H Begemann

**Affiliations:** Cognitive Neuroscience Center, University of Groningen, University Medical Center Groningen (UMCG), 9713AP Groningen, The Netherlands; Department of Languages, Literature and Communication, Institute for Language Sciences, Utrecht University, 3521JK Utrecht, The Netherlands; Department of Psychiatry, University Medical Center of Utrecht, University of Utrecht, 3584CS Utrecht, The Netherlands; Department of Neurology and Vrije Universiteit Brussel, UZ Brussel, 1090 Jette Brussel, Belgium; Department of Psychiatry, University Medical Center of Utrecht, University of Utrecht, 3584CS Utrecht, The Netherlands; Department of Psychiatry, Amsterdam UMC, 1105AZ Amsterdam, The Netherlands; Dimence Institute for Specialized Mental Health Care, Dimence Group, 7416SB Deventer, The Netherlands; Cognitive Neuroscience Center, University of Groningen, University Medical Center Groningen (UMCG), 9713AP Groningen, The Netherlands; Arkin, Institute for Mental Health, 1033NN Amsterdam, The Netherlands; Department of Psychiatry, University Medical Center of Utrecht, University of Utrecht, 3584CS Utrecht, The Netherlands; Altrecht, Mental Health Care Institute, 3562KX Utrecht, The Netherlands; Cognitive Neuroscience Center, University of Groningen, University Medical Center Groningen (UMCG), 9713AP Groningen, The Netherlands; Cognitive Neuroscience Center, University of Groningen, University Medical Center Groningen (UMCG), 9713AP Groningen, The Netherlands

**Keywords:** childhood trauma, childhood adversity, brain age, transdiagnostic, bipolar, psychosis

## Abstract

**Background and Hypothesis:**

Childhood trauma has been associated with accelerated biological aging, such as early pubertal development and cellular aging. Less is known about the impact of childhood trauma on brain aging, particularly across mental disorders. This cross-sectional study tested the hypothesis that childhood trauma severity is linked to accelerated brain aging using a large discovery MRI dataset. Another independent dataset was included to replicate findings.

**Study Design:**

The discovery dataset included 495 participants (248 bipolar-I, 126 schizophrenia-spectrum, 121 controls). To replicate findings, we used an independent dataset (*n* = 163; 80 schizophrenia-spectrum, 83 healthy individuals). Brain age gap estimate (BrainAGE; predicted brain age minus chronological age) was evaluated from T1-weighted MRI scans using pre-trained machine learning models to quantify brain aging, reflecting the difference between brain-predicted and actual age. Linear regressions were performed to assess the relationships between childhood trauma and BrainAGE across samples.

**Study Results:**

In the discovery dataset, higher childhood trauma severity was associated with increased BrainAGE (β = 0.09, *P* = .048), independent of diagnosis A categorical approach to trauma confirmed a dose–response pattern, with increased BrainAGE in individuals reporting multiple trauma types and across quartiles of cumulative trauma scores. The replication dataset showed similar results. The dimension of deprivation, but not threat, was associated with increased BrainAGE, in both datasets. Educational attainment moderated the effect of deprivation on BrainAGE in the discovery dataset only.

**Conclusions:**

This large transdiagnostic study suggests that individuals exposed to childhood trauma, especially deprivation, may undergo a process of accelerated brain aging, independent of psychiatric diagnosis.

## Introduction

Childhood trauma, such as abuse and neglect, can alter the pace of normal development by accelerating biological aging,^1^^,^^2^ which may disrupt psychological and social well-being.^3^^,^^4^ A meta-analysis of 54 studies (*n* = 116 010) linked early-life trauma to accelerated pubertal development and cellular ageing (including shorter telomere length and advanced DNA methylation age).^1^ Recently, childhood trauma has also been associated with accelerated physiological aging based on blood metrics (eg, C-reactive protein and white blood cell count)^5^^,^^6^ and facial aging.^7^

Less is known about the impact of childhood trauma on brain aging.^8^ The stress acceleration hypothesis posits that early-life trauma may speed up neurodevelopment as an adaption to a dangerous or unpredictable world.^8–11^ In a meta-analysis, Colich et al. found that early-life trauma was associated with cortical thinning across 25 neuroimaging studies (*n* = 3258).^1^ One limitation of traditional brain aging studies based on group-level differences is that they do not fully capture the individual brain aging process.^12^ Recent studies have applied machine learning models trained on neuroimaging data from healthy individuals along with their chronological age.^12^ These models can be applied to another dataset to predict individuals’ brain age, then compared to their chronological age, yielding a difference score: the brain age gap estimation (BrainAGE).^13^ Positive and negative BrainAGE values indicate accelerated (advanced) and delayed brain aging, respectively.^13^ This approach is increasingly used to characterize brain health,^14^ with increased BrainAGE commonly observed in schizophrenia-spectrum disorders,^15–17^ bipolar disorder,^15^^,^^16^ posttraumatic stress disorder,^18^ depression and anxiety.^19^

Studies are now starting to explore the link between childhood trauma and the brain age gap. So far, evidence is largely based on young and homogeneous samples, with conflicting results—positive, negative, or non-significant associations between early-life trauma and the brain age gap.^18–23^ As childhood trauma is a transdiagnostic risk factor for psychopathology, it is crucial to investigate its impact on the brain age gap using a transdiagnostic approach,^24^^,^^25^ and so it can be evaluated whether accelerated brain aging is a transdiagnostic mechanism linking childhood trauma to psychopathology. Understanding how early-life trauma contributes to advanced brain aging may clarify long-term vulnerability and underlying neurobiological mechanisms in mental health.

In this context, recent conceptual models highlight the importance of distinguishing between childhood experiences characterized by deprivation (absence of expected environmental inputs, eg, neglect and food insecurity) versus threat (experiences involving harm or its threat, eg, abuse and violence).^26^ Different experiences may yield different impact on neurodevelopment, cognitive functioning, and psychopathology.^27–30^ For example, deprivation-specific reductions have been found in total cortical volume,^31^ while threat-specific reductions were mostly seen in frontal gray matter (GM).^32^ Only three papers have investigated the impact of different dimensions of trauma on brain age gap, in youth samples only, with inconsistent results.^20^^,^^22^^,^^23^ Another underexplored factor is socioeconomic status (SES).^6^^,^^33^ Higher educational attainment, as a key indicator of SES, may act as a protective factor against accelerated aging, potentially delaying cognitive decline and increasing brain reserve in cognitively normal individuals.^34^ So far, no studies have investigated whether education can moderate the impact of childhood trauma on brain aging.

In this study, we investigated the association between childhood trauma and brain age gap. The primary (discovery) dataset included healthy individuals, and patients with bipolar disorder or schizophrenia-spectrum disorders, which share childhood trauma as an environmental risk factor.^35^^,^^36^ We used brain-age models trained to predict brain age from age-related brain patterns. Applying these models to our data yielded an estimated brain age for each participant. The difference between this estimate and chronological age is referred to as the BrainAGE. To our knowledge, this is the first study investigating these diagnostic categories in the context of childhood trauma and brain aging. We hypothesized: (1) greater trauma severity is associated with increased BrainAGE^21^; (2) both dimensions of deprivation^22^^,^^37^ and threat^20^ are associated with increased BrainAGE. Additionally, we explored whether education moderated any found associations. To replicate findings, we repeated analyses in a secondary (replication) dataset, comprising schizophrenia patients and healthy individuals.

## Methods

### Participants

#### Discovery Dataset

Similar to a previous MRI study from our group,^38^ we included participants from 3 studies: the Bipolar Genetics study,^39^ the Spectrum study,^40^ and the Simvastatin for Recent-Onset Psychosis study (baseline data).^41^ Participants were over 18 years of age. The final dataset included 121 healthy individuals, 126 patients with schizophrenia-spectrum disorders, and 248 patients with type I bipolar disorder.

#### Replication Dataset

The replication dataset was derived from the Genetic Risk and Outcome of Psychosis (GROUP) study,^42^ conducted at the University Medical Center Utrecht. This dataset comprised 83 healthy controls and 80 patients with schizophrenia (baseline data).

More detailed descriptions of the participant recruitment, inclusion/exclusion criteria, and clinical assessments are provided in the Supplementary Methods.

### Childhood Trauma and Educational Level

Childhood trauma was measured using the Dutch version of the Childhood Trauma Questionnaire–Short Form (CTQ-SF).^43^ This 25-item questionnaire assesses five trauma subtypes: emotional abuse, physical abuse, sexual abuse, emotional neglect, and physical neglect, summing up to a total trauma score. Established cut-off scores for moderate to severe trauma were applied to classify the presence of a specific trauma subtype: emotional neglect (≥15), physical neglect (≥10), emotional abuse (≥13), physical abuse (≥10), and sexual abuse (≥8). Following the dimensional model of trauma,^26^ we also calculated two composite trauma dimensions: the deprivation dimension (summing emotional and physical neglect), and the threat dimension (summing emotional, physical, and sexual abuse).

Regarding educational level, we converted the highest educational level achieved into the number of years of education (YoE, Table S1).

### Structural Magnetic Resonance Imaging

#### Discovery Dataset

High-resolution, three-dimensional T1-weighted images were obtained using a 3 T Philips Achieva scanner equipped with an 8-channel SENSE head coil (Philips, The Netherlands), details can be found in the Supplementary Method. The structural images were processed using FreeSurfer (v5.3.0).^44^ This automated pipeline performed segmentation and parcellation of cortical and subcortical structures based on the Desikan–Killiany atlas.^45^ This process yielded 68 measures of cortical surface area and cortical thickness, along with 14 subcortical volume measures.

#### Replication Dataset

In the replication dataset, T1-weighted images were acquired using a 1.5 T Philips Achieva scanner. Images were processed using a voxel-based morphometry pipeline.^46^ Partial volume gray and white matter segments were created, blurred using a 3-dimensional Gaussian kernel with a full width at half maximum of 8 mm to enhance statistical power, and resampled to voxel sizes of 2 × 2 × 2.4 mm^3^, and transformed to a standard space. The resulting blurred GM density maps, with voxel values between 0 and 1, reflected the local presence of GM tissue. More details can be found in Supplementary Methods.

### Brain Age Gap Estimates

In the discovery dataset, brain age values for male and female participants were estimated using a sex-specific, machine learning model developed by the ENIGMA-Lifespan working group.^47^ This model, trained and validated on data from over 30 000 healthy individuals, utilized a set of features derived from FreeSurfer output, including 68 cortical thickness measures, 68 cortical surface area measures, and 14 subcortical GM volumes. These features were used to predict brain age for each participant, with the model accounting for sex-specific variations and applying different models across specific age ranges. In the replication dataset, brain age values had been estimated previously based on the whole-brain GM density features, using a brain age prediction model that was trained on data from 386 healthy participants employing support vector regression.^17^

For both datasets, the brain age gap estimate (BrainAGE) was computed for each participant by subtracting chronological age from the predicted brain age. The processing flowchart can be found in Figure S1. Given that BrainAGE values are usually systematically overestimated in younger individuals and underestimated in older individuals (the so-called regression dilution), we applied a robust scheme to account for the effect of chronological age in both datasets.^48^

### Statistical Analysis

All statistical analyses were conducted using R (v4.1.1). All models included the following covariates: sex, YoE, group (dummy coding healthy controls, schizophrenia-spectrum disorder, and [for the discovery sample] bipolar type-I), and educational attainment. To account for the different scan parameters within the discovery dataset, we harmonized the data by including scan quality (high vs. low) as a covariate in all relevant statistical models.

First, we investigated the relationship between the cumulative trauma severity and BrainAGE using linear regression models, in both the discovery and replication dataset. Total childhood trauma score served as the independent variable, and BrainAGE was the dependent variable. Both unstandardized beta coefficients (*b*) and standardized coefficients (*β*) were reported. Follow-up analyses using a categorical trauma approach were conducted. Specifically, we applied the Jonckheere-Terpstra trend test to examine the effects of (1) the number of distinct trauma subtypes experienced by individuals, and (2) trauma severity quartiles, based on the distribution of total trauma scores (see Supplementary Methods for details). Additionally, we conducted an exploratory analysis by adding a sex × trauma interaction term to test whether associations with brain age gap differed by sex.

Second, to examine the effects of distinct trauma dimensions, we included deprivation and threat as independent variables in the same model, with BrainAGE as the dependent variable. Multicollinearity was assessed using the variance inflation factor (VIF), and no concern was observed (VIF < 10).

Third, in both datasets, we examined whether educational level moderated the relationship between childhood trauma and BrainAGE. We added the interaction term (total childhood trauma × YoE; deprivation scores × YoE; threat scores × YoE, respectively) in above models. The moderation analysis is particularly valuable for identifying subgroups in which the association between trauma and brain aging may differ, offering insights into specific populations that may be more vulnerable or resilient to the effects of childhood trauma on brain aging.

For the first 2 research aims, no correction for multiple testing was applied as the analyses were hypothesis-driven and evaluated in two independent datasets, providing a strong statistical framework. The third aim, testing the moderating role of education, was considered exploratory. To address concerns regarding the false discovery rate (FDR) in this set of analyses, we applied the Benjamini-Hochberg procedure across the three moderation models,^49^ setting the FDR at *q* = 0.05.

## Results

### Descriptive Statistics

In the discovery dataset of 495 participants, the mean BrainAGE was 1.02 years (SD = 5.70) (see Table 1). Specifically, the mean BrainAGE was 0.16 years (SD = 4.40) among 121 healthy controls, 0.84 years (SD = 5.31) in the 126 patients with a schizophrenia-spectrum disorder, and 1.53 years (SD = 6.38) in the 248 patients with bipolar disorder type I. In the replication dataset, the mean BrainAGE was -1.14 years (SD = 4.64) for 83 healthy controls and 2.06 years (SD = 5.63) for 80 patients with schizophrenia.

**Table 1:** Participant Characteristics

	Discovery dataset (*n* = 495)			Replication dataset (*n* = 163)	
	HC (*n* = 121)	SCZ (*n* = 126)	BP (*n* = 248)	HC (*n* = 83)	SCZ (*n* = 80)
Age, mean (SD), year	35. 34 (14.29)	31.00 (10.00)	48.06 (12.20)	27.90 (8.56)	26.8 (5.27)
Male, *N* (%)	62 (51.2%)	84 (66.7%)	119 (48%)	41 (49.4%)	65 (81.3%)
Education, mean (SD), year	14.43 (2.18)	13.23 (2.47)	13.49 (2.79)	12.60 (1.85)	10.80 (2.84)
Brain age gap, mean (SD), year	0.16 (4.40)	0.84 (5.31)	1.53 (6.38)	−1.14 (4.64)	2.06 (5.43)
Childhood trauma score	35.79 (11.29)	42.12 (14.26)	40.47 (12.13)	34.1 (5.17)	41.6 (11.0)
Childhood trauma numbers					
0, *N* (%)	92 (76.0%)	72 (57.1%)	126 (50.8%)	61 (73.5%)	41 (51.3%)
1, *N* (%)	15 (12.14%)	23 (18.3%)	62 (25.0%)	21 (25.3%)	23 (28.8%)
2, *N* (%)	4 (3.3%)	15 (11.9%)	35 (14.1%)	0 (0%)	6 (7.5%)
3 and 3 more, *N* (%)	10 (8.3%)	16 (12.7%)	25 (10.1%)	1 (1.2%)	10 (12.6 %)
Threat dimension score	19.48 (7.32)	22.84 (9.54)	19.89 (6.95)	16.8 (3.39)	21.1 (7.89)
Deprivation dimension score	16.31 (5.22)	19.28 (6.56)	19.36 (6.50)	17.4 (2.91)	20.5 (4.46)

Abbreviations: HC = healthy control; SCZ = schizophrenia; BP = bipolar.

### Total Childhood Trauma Severity and Brain Age Gap

Considering the total sample in the discovery dataset (*n* = 495), we found a significant association between increased childhood trauma severity and increased BrainAGE (*b* = 0.04, *β* = 0.09, *P* = .048), correcting for group (*b*_SCZ_ = 0.37, *P* = .64; *b*_BP_ = 0.42, *P* = .65) and including sex, YoE, and T1-scan quality as covariates. No group-by-trauma interactions were observed. In the replication dataset, the association between total trauma severity and BrainAGE was not significant (*n* = 163; *b* = 0.04, *β* = 0.06, *P* = .46, see Figure 1). Our supplementary Jonckheere-Terpstra tests using a categorical approach to trauma revealed that individuals exposed to an increasing number of trauma subtypes showed greater BrainAGE, as well as individuals with higher quartiles of cumulative trauma scores, which was confirmed in the replication dataset (see Supplementary Results and Figure S2). There was no significant interaction between childhood trauma and sex in predicting BrainAGE (see Supplementary Results).

**Figure 1:**
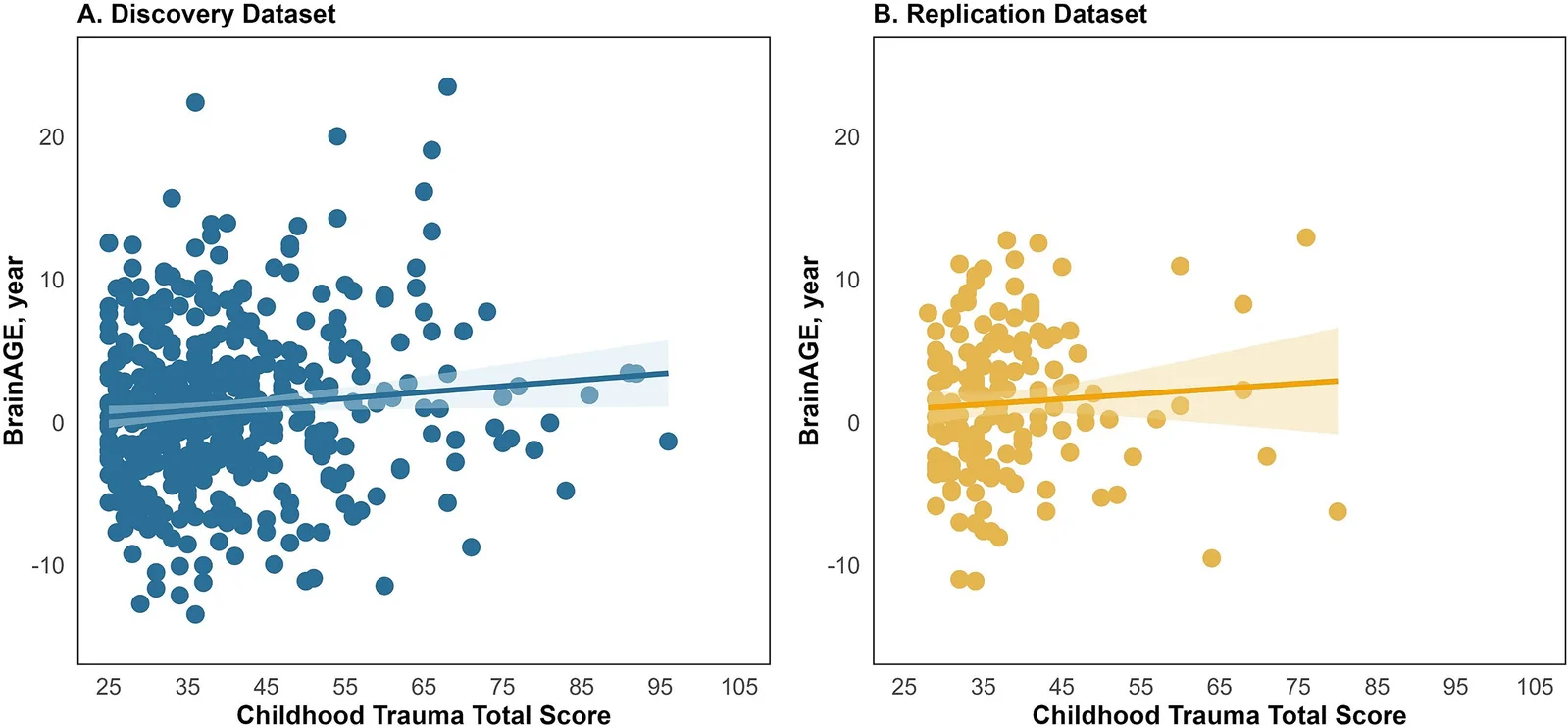
The Association Between Childhood Trauma and Brain Age Gap in Discovery Dataset (A) and Replication Dataset (B).

### Dimensions of Childhood Trauma and Brain Age Gap

In the discovery dataset, the deprivation dimension was significantly associated with increased BrainAGE (*b* = 0.11, *β* = 0.12, *P* = .03), while the threat dimension was not (*b* = −0.01, *β* = −0.01, *P* = .82) (Table 2). In the replication dataset, similar patterns were observed: deprivation was associated with an increased BrainAGE (*b* = 0.27, *β* = 0.21 *P* = .04), while the threat dimension was not (*b* = −0.09, *β* = −0.11, *P* = .25).

**Table 2:** Linear Regression Coefficients for the Associations Between Childhood Trauma Dimensions and Brain Age Gap

	**Discovery dataset (*n* = 495)**			**Replication dataset (*n* = 163)**		
	*b* (SE)^a^	*β* ^b^	*P-*value	*b* (SE)^a^	*β* ^b^	*P*-value
Intercept	2.69 (2.03)	NA	.19	−4.50 (4.00)	NA	.26
Threat	−0.01 (−0.01)	0.05	.82	−0.09 (0.08)	−0.11	.25
Deprivation	0.11 (0.05)	0.12	**.03**	0.27 (0.13)	0.21	**.04**
Education	−0.22 (0.10)	−0.10	**.03**	−0.35 (0.24)	−0.17	.05
Sex (female)	−0.75 (0.53)	−0.07	.16	1.31 (0.92)	0.12	.15
T1 scan quality	−0.79 (0.98)	−0.07	.42	NA	NA	NA
Group	SCZ: 0.38 (0.80)	0.03	.63	SCZ: 2.57 (0.93)	0.24	**.006**
	BP: 0.26 (0.93)	0.02	.78			

Abbreviations: SCZ = schizophrenia; BP = bipolar.

^a^ *b*: raw regression coefficients; SE: standardized error.

^b^ *β*: standardized regression coefficients. Bold *P* values indicate statistically significant results (*P* < 0.05).

### Role of Educational Attainment

In the discovery dataset, the interaction between total trauma and YoE was nonsignificant (*b* = −0.007, *β* = −0.17, *P*_FDR_ = .37), which was similar in the replication dataset (*b* = −0.01, *β* = −0.13, *P*_FDR_ = .25). For deprivation, the interaction with YoE remained significant after FDR correction (*b* = −0.03, *β* = −0.41, *P*_FDR_ = .03). Simple slope analysis showed stronger and significant associations between deprivation and BrainAGE in individuals with low (*b* = 0.13, 95% CI, 0.05-0.21) and moderate education levels (*b* = 0.10, 95% CI, 0.01-0.18), compared to those with high education, where the association was weaker and non-significant (*b* = 0.05, 95% CI, –0.05 to 0.15) (see Figure 2). However, this interaction effect was not observed in the replication dataset (*b* = −0.02, *β* = −0.12, *P*_FDR_ = .86).

**Figure 2:**
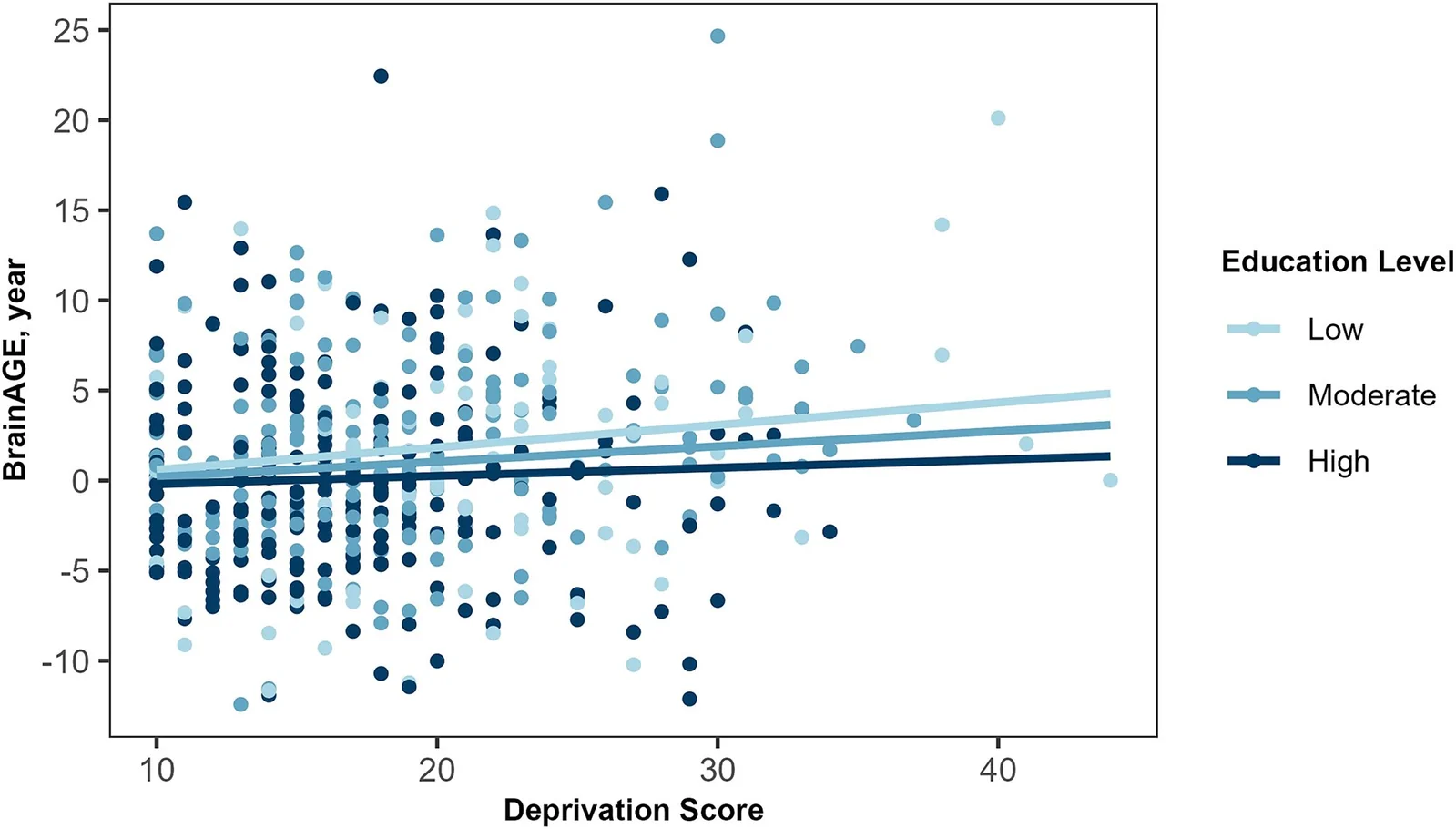
Moderating effect of educational attainment on the association between childhood deprivation and brain age gap in the discovery dataset. Abbreviation: BrainAGE = brain age gap estimate. The interaction effect model between deprivation and education level was adjusted the effect of sex, T1 scan quality, and diagnosis group. The effect of deprivation varied at different education levels (low: <12 years; moderate: 12-14 years; high: >14 years).

## Discussion

In this study, we used a transdiagnostic approach to investigate the link between childhood trauma and brain aging. In our primary dataset, we discovered that higher childhood trauma severity was associated with a BrainAGE of 0.04 year per point on the childhood trauma severity, independent of diagnosis. Supplementary ranked analyses confirmed increased BrainAGE in individuals with multiple trauma subtypes and higher severity quartiles, which was also replicated in the second dataset. We discovered and replicated that deprivation-related trauma (neglect) was associated with increased BrainAGE, while threat-related trauma (abuse) was not. Educational attainment moderated this association in the discovery sample, with stronger effects among individuals with low and moderate education; this effect was absent in the replication dataset.

Current findings extend the emerging literature on the impact of childhood trauma on brain age gap, showing both linear and categorical dose–response relationships in our discovery dataset, independent of diagnosis. This aligns with a previous study, which reported an association between childhood trauma and increased BrainAGE in 150 adolescents, utilizing a broad trauma index including both childhood trauma and socioeconomic adversities.^21^ Another study of 10 000+ adolescents also linked early trauma and family aggression to advanced brain age.^20^ However, this study mainly evaluated objective traumatic events (eg, accidents, disasters, and sudden family member death), which may differ in scope or impact from the more personal or family-level trauma (eg, emotional and physical abuse, neglect) as examined in our study. Two prior studies in adult samples that focused on childhood trauma and brain age gap did not observe a significant association. One involved both patients with PTSD (*n* = 882) and healthy individuals (*n* = 1347),^18^ while the other was a smaller transdiagnostic study (*n* = 285) including patients with depression or anxiety disorders and healthy controls.^19^ Additional large-scale transdiagnostic studies are needed to investigate the extent to which the so-called p-factor,^50^^,^^51^ reflects the general and independent impact of childhood trauma.

As hypothesized, we discovered and replicated a positive association between the dimension of deprivation and increased brain age gap, suggesting that exposure to deprivation-related adversity, including different types of neglect, has a global effect by accelerating whole-brain aging. Keding et al.^22^ also found that physical neglect was linked to a positive whole-brain brain age gap in a sample of 234 adolescent girls. In contrast, Beck et al. showed that emotional neglect was associated with a reduced brain age gap and therefore delayed brain maturation, in a large sample of 10 000+ children aged 9-11.^20^ The authors explain this discrepant findings by noting that their study evaluated lack of parents’ supervision and family/community support, while prior studies focused on more extreme forms of neglect or institutionalization. Against our expectations, no association between threat and brain age gap was found in either dataset. Keding et al. observed that childhood abuse (threat) corresponded with a reduced brain age gap (ie, delayed brain maturation), particularly within the emotion-related circuits.^22^ This process may explain our current non-findings for threat-related effects.

A differential impact between deprivation versus threat-related experiences in shaping brain ageing trajectories, may be explained by the observation that brain development during childhood and adolescence is highly dynamic. After initial growth, a period of cortical pruning sets in, during which the cortex becomes thinner, while the cortical surface still expands.^52^ Somewhat later in adolescence the cortical surface area starts decreasing too, while the white matter continues to mature, in some brain areas even during young adulthood.^53^ While this pattern of neurodevelopment holds for typically developing children as a group, there are large individual differences in the size and timing of the changes.^54^ In case of deprivation, the absence of age-typical social and cognitive stimuli may stimulate the global acceleration of neuronal and synapse elimination mechanisms typical for low-complexity environments.^22^^,^^26^^,^^55^ Indeed, it has been described that insufficient cognitive and social stimulation may lead to fewer brain connections, making the brain more prone to age-associated volume decline.^56^ In contrast, as also speculated by Keding et al., threat may be associated with a delay in normal age-appropriate GM reductions in emotion circuits.^22^ This delayed structural maturity may reflect a state of generalized threat hypervigilance, which is normally observed only in younger children. Such an enhanced threat bias in neurobiological reactivity can be adaptive in abusive environments. Previous studies have indeed linked threat generalization to increased recruitment of threat processing circuits with maltreatment exposure and an increased risk for psychopathology.^57^^,^^58^ As childhood trauma may accelerate or decelerate brain development, depending on its dimension,^26^ timing,^59^ and interval between exposure and age of assessing, it is crucial that further research aims to measure all these aspects of (brain) development in large samples of both typically developing children,^60^ and children at (family) risk of developing psychotic or bipolar disorders.^61^

Furthermore, our study reveals that educational attainment may moderate the association between accelerated brain aging and childhood deprivation in specific. The effect of childhood deprivation on brain aging seems significantly more pronounced in individuals with lower educational attainment than those with higher levels, suggesting that education may buffer trauma’s effect on brain health. Education is closely tied to SES, and prior studies have shown accelerated brain aging in individuals from low-SES backgrounds.^33^^,^^62^ The relationships among childhood trauma, educational attainment, SES, and mental health are complex and dynamic, involving crucial developmental processes such as emotion regulation, cognitive function, cognitive reserve and attachment, as well as interactions among various ecological systems including family and schools, and a bidirectional link between academic achievements and mental health.^63^ Our findings support the notion that higher educational attainment is associated with reduced adverse effects of childhood trauma on brain aging in adulthood. One possible explanation is that individuals with more education may have experienced environments that fostered enhanced cognitive stimulation,^64^ additional emotional support from school,^65^ but also a healthier lifestyle.^66^ Recent evidence suggests education may reflect early brain advantages (eg, larger brain volume) rather than causally protect the brain.^67^ Because these findings are based on large-scale samples older than 50 years old, the role of education during development remains unclear. While programs promoting educational attainment, such as mentoring program, may support breaking the cycle of trauma and fostering long-term brain health,^68^ a causal effect during development is still lacking.

Strengths of current study include its transdiagnostic design and the use of machine learning to associate childhood trauma with brain age gap. By utilizing 2 independent datasets, we aimed to test and validate (replicate) this association. One important limitation is that our smaller replication dataset probably lacked power to detect a trauma-related accelerated brain aging as observed in the discovery dataset. For our categorical approach, our replication dataset confirmed accelerated brain aging in those with multiple trauma types and higher cumulative trauma scores. Second, 2 different data preprocessing pipelines were applied for the discovery and replication datasets (surface-based vs. volume-based, respectively), limiting comparability. The generated data was applied into the two different brain age prediction models. The average BrainAGE in healthy controls in the replication dataset deviated from the expected mean of zero (–1.14 years), potentially reflecting methodological differences. This methodological heterogeneity limits the interpretation of our findings and warrants replication. However, the associations between BrainAGE and trauma were consistently observed across these divergent approaches may suggest that the trauma-related BrainAGE differences are not specific to one particular pipeline or algorithm, which could strengthen the robustness of our findings. Third, using T1-based brain age models may not capture all aspects of brain aging; future research could improve precision by integrating T2, DTI, and rs-fMRI imaging. Fourth, our analysis focused on the global brain age gap, lacking specificity regarding brain networks or circuits. Fifth, we did not account for factors such as trauma timing, recent stressors, illness duration, medication use, genetic influences, or other potential confounders like social isolation.^69^ Finally, cross-sectional and retrospective data may introduce recall bias, limiting causal conclusions. Future research should prioritize longitudinal cohort studies, analyze large-scale transdiagnostic datasets from international consortia, and incorporate genetic and environmental risks along with gene–environment correlations.^70^ It may also be mentioned that current study did not include complementary (bio)markers such as cognitive assessments, inflammatory profiles, or epigenetic age indices.^71–73^ Including such measures could provide convergent validation of brain age estimates, and may help to elucidate the biological and functional correlates of brain aging in trauma-exposed individuals.^74–77^ Future studies using a multimodal approach will be essential for deepening our understanding of the mechanisms underlying early brain aging. Furthermore, we found no sex differences in the trauma–brain age gap association, possibly due to limited statistical power. Although sex was included as a covariate in our primary analyses, future work should investigate whether trauma-related brain aging differs by sex or gender, given evidence for sex differences in both trauma exposure and brain development.^78^

In summary, our findings show that severity of childhood trauma, particularly deprivation-related trauma, is associated with accelerated brain aging, independent of diagnosis. Education moderated this association, underscoring the its potential importance for brain health.

## Supplementary Material

Supplement_mr_sbaf204
