# Supplementary material for: The Transdiagnostic Association Between Childhood Trauma and Accelerated Brain Aging in Two Independent Datasets
Source: Schizophr Bull. 2025 Nov 23;52(5):sbaf204. doi: 10.1093/schbul/sbaf204 (PMC13645823; doi:10.1093/schbul/sbaf204)
Supplement: Supplementary file 1 [file supplement_mr_sbaf204.docx]

**Supplement**

**Methods**

***Sample***

Discovery Dataset

A total of 495 subjects were recruited as part of three different studies, conducted in the UMCU, the Netherlands: *Bipolar Genetics study*, *Spectrum* *study* and *Simvastatin for recent-onset psychosis.* All participants were ≥18 years of age. Healthy control subjects did not have a current or past psychiatric diagnosis. Patients met Diagnostic and Statistical Manual for Mental Disorders, Fourth Edition (DSM-IV; American Psychiatric Association, 2000) criteria for bipolar disorder type-I or a schizophrenia spectrum disorder. Studies were approved by the relevant medical ethical committee, all participants gave written informed consent.

The *Bipolar Genetics study* investigated genetic and phenotypic information of patients with bipolar disorder type-I, first-degree relatives and controls ^1^. For the current study, we included 248 bipolar type-I patients who were recruited via clinicians, the Dutch patients’ association, pharmacies and advertisements. Presence or absence of psychopathology was established using the Structured Clinical Interview for the Diagnostic and Statistical Manual of Mental Disorders DSM-IV.

The *Spectrum* *study* examined psychotic experiences across the continuum of patients with a psychotic disorder and the general population. We included data from 42 patients with a schizophrenia spectrum disorder who were enrolled via clinicians and the Dutch patients’ association. Healthy controls were recruited by means of a website ‘www.verkenuwgeest.nl’ (‘explore your mind’, see ^2^. Visitors of this Website filled out a self-report questionnaire that is designed to quantify the tendency to hallucinate in healthy individuals (modified version of the Launay and Slade Hallucination Scale [LSHS]), they were subsequently invited to visit the UMCU. Psychiatric diagnosis (in patients) or the absence of psychopathology (in healthy individuals) was evaluated using the Comprehensive Assessment of Symptoms and History Interview (CASH, ^3^. Of the 121 healthy individuals included in the discovery dataset, 37 reported auditory verbal hallucinations (at least once a month, for more than 1 year). These individuals are regarded as healthy individuals since the presence of a psychotic disorder had been ruled out by a psychiatrist, as they did not show professional, psychological or social dysfunction.

*Simvastatin for recent-onset psychosis* was a medication trial ^4^, investigating the augmenting effects of simvastatin treatment (40 mg/daily) versus placebo. Participants used study medication for one year and visited the UMCU approximately once every three months for study measurements. We included baseline data for 84 patients with a diagnosis of a schizophrenia spectrum disorder, who had been recruited via collaborating health institutions. Psychiatric diagnosis was confirmed using the Comprehensive Assessment of Symptoms and History Interview (CASH, Andreasen et al., 1992). We also included baseline data for 39 healthy controls.

Replication Dataset

The Genetic Risk and Outcome of Psychosis (GROUP) study ^5^, is a longitudinal study with a focus on gene–environment vulnerability and resilience in patients, their unaffected family members and non-related controls, which offers the opportunity to elucidate etiological and pathogenetic factors influencing the onset and course of psychotic disorders. We included 83 healthy controls and 80 patients diagnosed with schizophrenia, which were recruited in the University Medical Center Utrecht, the Netherlands. All from the baseline assessment of the GROUP study. Eligible patients had to fulfill the following criteria: (1) age between 16 and 50 years, (2) meeting Diagnostic and Statistical Manual of Mental Disorders, Fourth Edition (DSM-IV) criteria for a nonaffective psychotic disorder (including schizophrenia, schizophreniform disorder, and schizoaffective disorder), (3) fluent in Dutch, and (4) able and willing to give written informed consent.

***Structural magnetic resonance imaging***

**Discovery Dataset**: All participants were scanned using the same MRI scanner at the University Medical Center Utrecht. Structural MRI scans of the whole brain were acquired using a 3T Philips Achieva scanner equipped with an 8-channel SENSE head coil (Philips, The Netherlands). High-resolution, three-dimensional T1-weighted images were obtained using the following parameters: 200 contiguous sagittal slices, echo time (TE) = 4.6 ms, repetition time (TR) = 10 ms, flip angle = 8°, field of view (FOV) = 240 mm, with a voxel size of 0.75 × 0.75 × 0.80 mm³. For a subset of 51 participants, lower-resolution T1-weighted images were acquired, with 160 contiguous sagittal slices, TE = 4.6 ms, TR = 10 ms, flip angle = 8°, FOV = 224 mm, and a voxel size of 1 × 1 × 1 mm³. All scans were reviewed by a radiologist, blinded to participant status, to check for any radiological abnormalities. To account for the different scan parameters within this dataset, we harmonized the data by including scan quality (high vs. low) as a covariate in all relevant statistical models.

**Replication Dataset**: In the replication dataset, T1-weighted images were acquired using a 1.5T Philips Achieva scanner, with a resolution of 1 × 1 × 1.2 mm³. Three-dimensional coronal spoiled-gradient echo scans were obtained with the following parameters: matrix size of 256 × 256, TE = 4.6 ms, TR = 30 ms, flip angle = 30°, and 160-180 contiguous slices with voxel size of 1 × 1 × 1.2 mm³ and FOV = 256 mm.

***Statistical analysis***

To further explore any significant findings in both datasets, we applied a categorical approach to account for notable patterns in trauma scores, particularly given the low trauma scores in healthy controls and the significant proportion of patients reporting trauma. We utilized the Jonckheere-Terpstra (JT) test for ordered alternatives (one-tailed) (Jonckheere, 1954). For clinical relevance, participants were grouped based on the number of moderate to severe trauma subtypes, resulting in four categories: no trauma, one trauma subtype, two trauma subtypes, and three or more trauma subtypes. Additionally, to address potential concerns regarding predefined cutoff values, we divided participants into quartiles based on the distribution of cumulative childhood trauma scores.

**Figure S1. Flowchart of data processing steps for discovery dataset (A) and replication dataset (B)**

Abbreviations: HC, healthy control; SCZ, schizophrenia, BP, bipolar.

(A) In the discovery dataset, the raw T1-weighted MRI scan from subject X was processed using FreeSurfer’s surface-based pipeline. From the output, 68 cortical surface area measures, 68 cortical thickness measures, and 14 subcortical volume measures were extracted and input into a pre-trained brain age prediction model (see Yu et al., 2024),^6^ to estimate brain age. Brain age gap was calculated as predicted brain age minus subject X’s chronological age. (B) In the replication dataset, the raw T1-weighted MRI scan from subject X was processed using a volume-based pipeline. The resulting gray matter density map for each subject was entered into a pre-trained brain age prediction model (see Schnack et al., 2016),^7^ to estimate brain age. Brain age gap was again calculated as predicted brain age minus chronological age.

Notes * Brain age gap values from the two pipelines were further adjusted for the effect of chronological age using the robust framework of Beheshti et al. (2019).^8^ The adjusted values were then used in the statistical analyses.

**Supplementary Table S1. Conversion values of educational levels.**

| **Educational level (CASH-III)** | **Years of education** |  |
| --- | --- | --- |
| None | 0 |  |
| Primary school | 6 |  |
| Lower secondary education/domestic science school | 10 |  |
| Middle general secondary vocational education | 10 |  |
| Higher general secondary education | 11 |  |
| Pre-university education | 12 |  |
| Community college/intermediate vocational education | 14 |  |
| Higher vocational education | 15 |  |
| Academic education | 17 |  |
| CASH-III, Comprehensive Assessment of Symptoms and History part III Andreasen et al., 1992) | | |

**Results**

Categorical Patterns of Trauma-Related Brain Age Accelerations

In the discovery dataset, individuals exposed to an increasing number of trauma subtypes exhibited an advanced BrainAGE (JT=39221, p=0.04). Cumulative trauma scores, independent from trauma subtype, revealed a similar dose-response pattern: higher quartiles of cumulative trauma scores were associated with a more advanced BrainAGE (Jonckheere-Terpstra test, JT=50320, p=0.005). These dose-response patterns were also observed in the replication dataset, where participants reporting multiple trauma types exhibited a more advanced BrainAGE (JT=3749, p=0.01), as did participants in higher trauma quartiles (JT=6090, p<0.001), see Figure S2.

The Interaction Effect of Childhood Trauma and Sex

The interaction effect between childhood trauma and sex was not significant in our discovery sample, b = -0.04, β = -0.16, p = 0.29. In the replicate sample, the interaction effect was not significant either, b = 0.06, β = 0.21, p = 0.50.

**Figure S2. Categorical patterns of childhood trauma and brain age gap in discovery dataset (A) and replication dataset (B)**

**Reference**

1.	Vreeker A, Boks MPM, Abramovic L, et al. High educational performance is a distinctive feature of bipolar disorder: a study on cognition in bipolar disorder, schizophrenia patients, relatives and controls. *Psychological Medicine*. 2016;46(4):807-818. doi:10.1017/S0033291715002299

2.	Sommer IE, Daalman K, Rietkerk T, et al. Healthy Individuals With Auditory Verbal Hallucinations; Who Are They? Psychiatric Assessments of a Selected Sample of 103 Subjects. *Schizophrenia Bulletin*. 2010;36(3):633-641. doi:10.1093/schbul/sbn130

3.	Andreasen NC, Flaum M, Arndt S. The Comprehensive Assessment of Symptoms and History (CASH): An Instrument for Assessing Diagnosis and Psychopathology. *Archives of General Psychiatry*. 1992;49(8):615-623. doi:10.1001/archpsyc.1992.01820080023004

4.	Begemann MJH, Schutte MJL, Slot MIE, et al. Simvastatin augmentation for recent-onset psychotic disorder: A study protocol. *BBA Clinical*. 2015;4:52-58. doi:10.1016/j.bbacli.2015.06.007

5.	Korver N, Quee PJ, Boos HBM, Simons CJP, de Haan L, GROUP investigators. Genetic Risk and Outcome of Psychosis (GROUP), a multi-site longitudinal cohort study focused on gene-environment interaction: objectives, sample characteristics, recruitment and assessment methods. *Int J Methods Psychiatr Res*. 2012;21(3):205-221. doi:10.1002/mpr.1352

6.	Yu Y, Cui H, Haas SS, et al. Brain‐age prediction: Systematic evaluation of site effects, and sample age range and size. *Hum Brain Mapp*. 2024;45(10):e26768. doi:10.1002/hbm.26768

7.	Schnack HG, van Haren NEM, Nieuwenhuis M, Hulshoff Pol HE, Cahn W, Kahn RS. Accelerated Brain Aging in Schizophrenia: A Longitudinal Pattern Recognition Study. *AJP*. 2016;173(6):607-616. doi:10.1176/appi.ajp.2015.15070922

8.	Beheshti I, Nugent S, Potvin O, Duchesne S. Bias-adjustment in neuroimaging-based brain age frameworks: A robust scheme. *NeuroImage: Clinical*. 2019;24:102063. doi:10.1016/j.nicl.2019.102063
